# Supplementary material for: Exploring the limits of hierarchical world models in reinforcement learning
Source: Sci Rep. 2024 Nov 6;14:26856. doi: 10.1038/s41598-024-76719-w (PMC11538428; doi:10.1038/s41598-024-76719-w)
Supplement: Supplementary file 1 — Supplementary Information. [file 41598_2024_76719_MOESM1_ESM.pdf]

## Supplementary Material

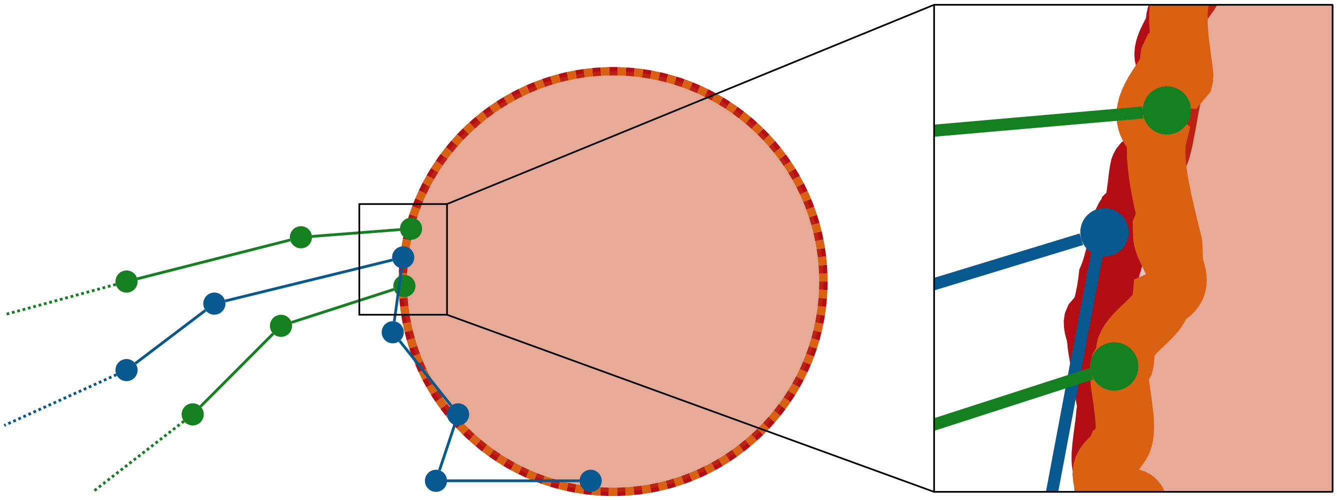

**Figure S1.** A model exploitation scenario in the Nav2d environment provoked by the level 1 agent, visualised via level 0 observations. **Left:** Three trajectories leading to the terminal region of the Nav2d environment. The large red-orange circle marks the terminal region, where the agent obtains at the same time a reward of 1. The two green trajectories are generated from temporally abstracted ground truth data, the blue trajectory is an open loop rollout of the level 1 RMA. It shows the jittering behaviour found by manual inspection of the agent environment interaction and the level 1 RMA training. **Right:** The border of the terminal region up-close. The level 1 world model is not completely accurate here outside of the regions trained via ground truth data. The reward border (red line) at which the model starts to predict positive reward is not everywhere perfectly aligned with the termination border (orange) at which the model starts to predict a high episode termination probability. This mismatch between the borders of the two regions, which should actually be perfectly superimposed, can be expected from the world model as it is not trained with ground truth data everywhere. At the same time, it is a reasonable assumption that over time the level 1 RMA finds these inaccuracies and exploits them for high reward.
